# Post-separation abuse: A concept analysis

**DOI:** 10.1111/jan.15310

**Published:** 2022-05-27

**Authors:** Kathryn J. Spearman, Jennifer L. Hardesty, Jacquelyn Campbell

**Affiliations:** 1 Johns Hopkins School of Nursing, Baltimore, Maryland, USA; 2 University of Illinois Urbana-Champaign, Champaign, Illinois, USA

**Keywords:** concept analysis, custody, divorce, intimate partner violence, nursing, post-separation abuse

## Abstract

**Aim:**

To report an analysis of the concept of post-separation abuse and its impact on the health of children and adult survivors.

**Design:**

Concept analysis.

**Data Sources:**

A literature search was conducted via PubMed, Cochrane and Embase and identified articles published from 1987 to 2021.

**Methods:**

Walker and Avant's (2019) eight stage methodology was used for this concept analysis, including identifying the concept, determining the purpose of analysis, identifying uses of the concept, defining attributes, identifying a model case and contrary case, antecedents and consequences and defining empirical referents.

**Results:**

Post-separation abuse can be defined as the ongoing, willful pattern of intimidation of a former intimate partner including legal abuse, economic abuse, threats and endangerment to children, isolation and discrediting and harassment and stalking. An analysis of literature identified essential attributes including fear and intimidation; domination, power and control; intrusion and entrapment; omnipresence; and manipulation of systems. Antecedents to post-separation abuse include patriarchal norms, physical separation, children, spatiality and availability, pre-separation IPV and coercive control and perpetrator characteristics. Consequences include lethality, adverse health consequences, institutional violence and betrayal, such as loss of child custody and economic deprivation.

**Conclusion:**

This concept analysis provides a significant contribution to the literature because it advances the science for understanding the phenomenon of post-separation abuse. It will aid in developing risk assessment tools and interventions to improve standards of care for adult and children survivors following separation from an abusive partner.

**Impact:**

This concept analysis of post-separation abuse provides a comprehensive insight into the phenomenon and a theoretical foundation to inform instrument development, future research and intervention. Post-separation abuse is a complex, multi-faceted phenomenon that requires differential social, legal and healthcare systems responses to support the health and well-being of survivors and their children.

## 1 ∣ INTRODUCTION

Intimate partner violence (IPV) is more prevalent among couples with children, as 60% of couples experiencing IPV have children living in the household (Hamby et al., 2011; McDonald et al., 2006; Rezey, 2020). Separation from an abusive partner is often thought to be the solution to ending violence; yet, abuse and the risk for lethality often escalates following separation (Campbell et al., 2003; Stark & Hester, 2019; Zeoli et al., 2013). Although all genders experience abuse, abuse towards women by their male partners following separation is enabled by patriarchal norms and is more lethal. Women are 10 times more likely to be victims of IPV than men, especially when abuse occurs after separation (Hardesty, 2002). Gender differences in economic power (wage disparities between partners), gendered discourses of parenting that undervalue mothers' unpaid domestic labour, and misogynistic norms that position mothers as obstructive or vindictive make mothers more vulnerable to post-separation abuse (Elizabeth et al., 2012). Most of the international research on post-separation abuse has focused on male perpetration of abuse towards the mothers of their children. For these reasons, we refer throughout this concept analysis to women, mothers and survivors.

Women who are separated and divorced report higher rates of IPV than married women; however, much of this research is cross sectional, and thus impossible to tell if separation occurred before or after the IPV. A 2010 report based on the National Crime Victimization Survey in the United States identified rates of IPV 30 times higher for separated women and nine times higher for divorced women as compared with married women based on 2-year rolling averages of reports of the prior 6 months (Catalano, 2012; Rezey, 2020; Toews & Bermea, 2017). After separation from an abusive partner, up to 90% of women report continued harassment, stalking or abuse (Davies et al., 2009; Hardesty et al., 2012; Mitchell et al., 2021). Yet, patterns of abusive behaviours following separation have not been clearly defined. Post-separation abuse is often missed by quantitative measures (Anderson & Saunders, 2003), especially the more covert types of abuse that arise following separation (Galántai et al., 2019; Lynch et al., 2021; Miller & Smolter, 2011). Few quantitative studies have been conducted that elucidate abusive tactics post-separation that include using children, threats, manipulation of visitation and co-parenting schedules, and withholding child support (Toews & Bermea, 2017) and how these tactics impact the health and well-being of children and families (Broughton & Ford-Gilboe, 2017).

A concept analysis of post-separation abuse is needed to develop a clear definition to accurately measure the phenomenon. Post-separation abuse is perpetrated at the individual level but facilitated and perpetuated by factors at the family (power differentials between intimate partners, stigma), community (legal system responses) and societal level (gender and patriarchal norms). IPV, including post-separation abuse, must be understood through the assaults on the personhood, dignity, autonomy, liberty and self-worth of the human being, and not just in terms of the physical bruises it leaves (Scheper-Hughes & Bourgois, 2004; Silverman et al., 2004; Stark & Hester, 2019). Following Walker and Avant's (2019) method of concept analysis, we outline the significance of the concept, followed by identifying its uses, the defining attributes, identifying a model and contrary case, antecedents and consequences, and empirical referents. In addition, we discuss limitations and implications for nursing.

## 2 ∣ BACKGROUND AND SIGNIFICANCE OF POST-SEPARATION ABUSE

Systems of care are currently geared towards helping individuals leave abusive relationships, even with the recognition that separation is a well-established risk factor for lethality for women and children (Campbell et al., 2009; Sillito & Salari, 2011). Approximately 1700 women are murdered by intimate partners per year in the US, bereaving an estimated 3300 children annually (Lewandowski et al., 2004; VPC, 2021). Estimating from the Campbell et al. (2003) 12-city intimate partner femicide study, approximately 44% of those women were separated from their partners when killed. Parental IPV, separation and custody disputes are risk factors for child homicides (Jaffe et al., 2012; Lucas et al., 2002; Lyons et al., 2021).

For parents with minor children, legal systems and policies that regulate divorce, separation and custody are the central context influencing the ability to maintain safety following separation from an abusive partner (Broughton & Ford-Gilboe, 2017; Hardesty & Chung, 2006; Jaffe et al., 2008; Saunders, 2007; Wuest et al., 2006). Yet, the family court context in and of itself creates conditions for abusive behaviours to arise following separation and divorce. The majority of high conflict custody cases involve IPV (Jaffe et al., 2008; Jaffe & Crooks, 2004). The divorce and custody literature that guides family court judicial decision-making frames conflict as mutual, which fails to account for the power and control dynamics of abuse. This framework is also damaging as it shifts the focus away from batterers' damaging behaviours and places blame on those experiencing abuse (Feresin et al., 2018). How violence is framed has significant implications for how it is addressed.

Mothers experiencing IPV face barriers to safety post-separation because they must negotiate co-parenting arrangements and family court (Broughton & Ford-Gilboe, 2017; Hardesty & Chung, 2006; Spearman et al., 2022; Stark & Hester, 2019). IPV, child maltreatment and children's exposure to IPV are frequently minimized or underdetected in family court proceedings, which has lasting consequences for survivors (Khaw et al., 2021; Meier, 2020; Saunders, 2015). Understanding specific tactics of post-separation abuse is crucial to designing interventions that acknowledge experiences in negotiating violence, separation and divorce, and the structural contexts that are barriers to safety and health.

## 3 ∣ METHODS

**FIGURE 1:**
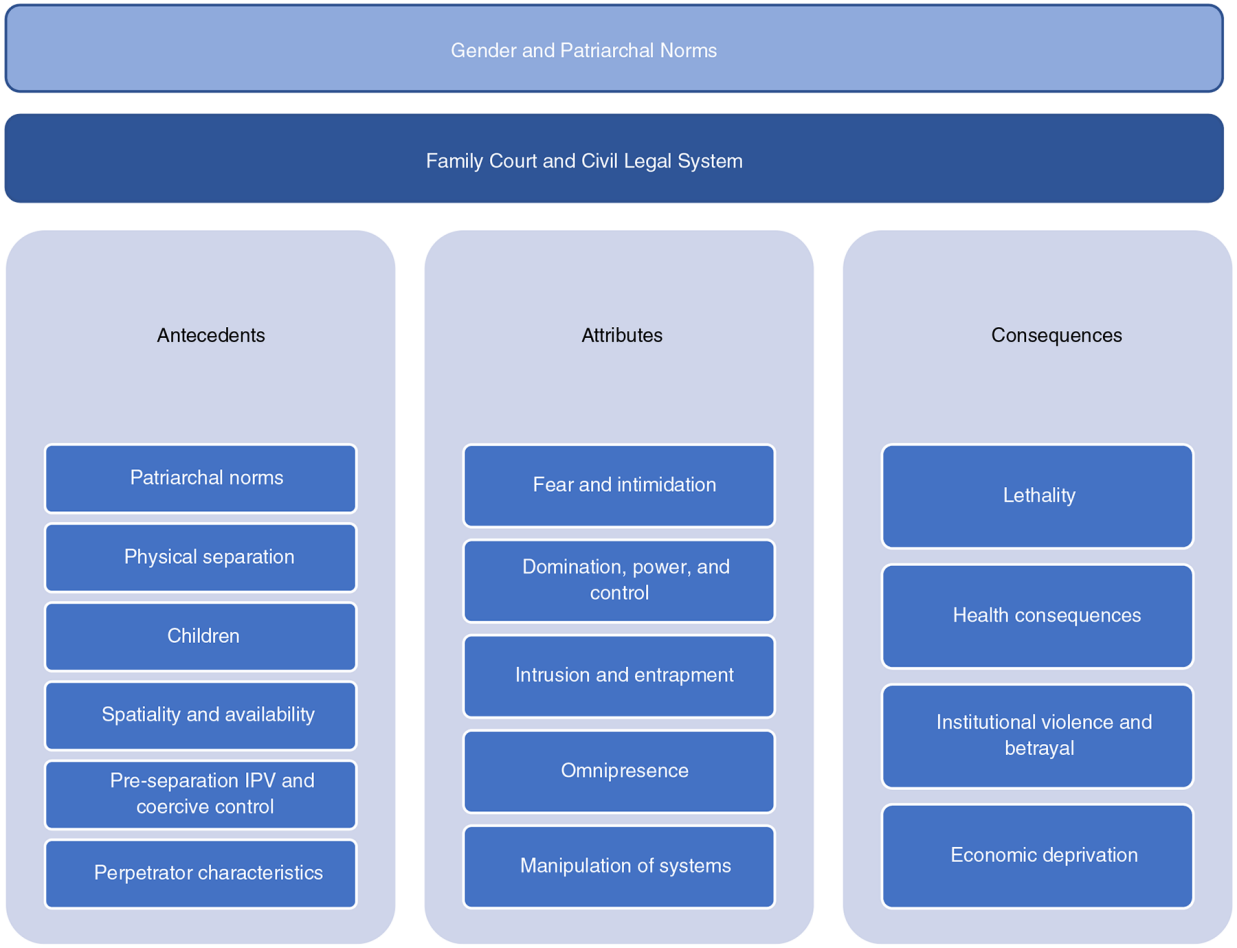
A concept analysis of post-separation abuse.

We employed Walker and Avant's (2019) eight step procedure of concept analysis, which is a systematic way to promote understanding and to develop a definition that will allow for measurement of the phenomenon. After selecting the concept of post-separation abuse, we determined the aims of the analysis. We conducted a review of the literature and identified the uses of the concept in disciplines including nursing, social work, psychology, family science, criminology and law. We examined the meaning of post-separation abuse, determined the defining attributes, identified a model and contrary case, antecedents and consequences (Figure 1), and empirical referents. We explored post-separation abuse through a literature search of PubMed, CINAHL PLUS and Embase using keywords including: ‘post-separation abuse’, ‘post-separation violence’, ‘post-separation assault’, ‘estrangement violence’, ‘separation violence’, ‘intimate partner violence’ AND ‘separation’, ‘intimate partner violence’ AND ‘coparenting’, ‘intimate partner violence’ AND ‘custody’, ‘separation’ AND ‘victimization’. The first author conducted the literature search and screened the articles, which resulted in 855 studies for screening, of which 612 were excluded because they did not address abusive behaviours that arise in the post-separation context. A further full text review of these 243 publications, resulted in excluding 109 manuscripts. Of these, we included 134 publications for this concept analysis, plus six studies identified in references of included manuscripts. In total, we identified 140 publications published from 1987 through 2021 (Appendix 1).

The purpose of this concept analysis is to enhance the understanding of the concept of post-separation abuse and its practical implications and provide a foundation for measurement and trans-disciplinary work to develop differential system responses.

## 4 ∣ IDENTIFYING AND DEFINING POST-SEPARATION ABUSE

Post-separation abuse can be defined as the ongoing, willful pattern of intimidation of a former intimate partner that includes (1) legal abuse, (2) economic abuse, (3) threats and endangerment to children, (4) isolation and discrediting and (5) harassment and stalking (Breiding et al., 2015; Brownridge, 2006; Dekerseredy et al., 2017; Godfrey & Robinson, 2014; Logan et al., 2008; Miller & Smolter, 2011; Sheridan, 2001; Walker et al., 2004; Zeoli et al., 2013). Post-separation abuse has also been termed ‘post-separation violence’, ‘separation or divorce assault’ or ‘estrangement violence’. Post-separation abuse is aligned theoretically with descriptions in the literature of intimate partner terrorism (Johnson, 2005) and coercive control (Stark & Hester, 2019), whereby violent and nonviolent tactics are used to wholly dominate an intimate partner and deprive them of free will. This contrasts with what is called situational couple violence, in which violence erupts out of specific arguments or conflicts but without an ongoing motive to dominate one's partner (Hardesty et al., 2012; Johnson, 2005).

Separation is a complex process, often involving iterations of leaving and returning. To operationalize ‘post-separation’ throughout this concept analysis, we focus on physical or legal separation (moving out, transitioning children between households or invoking some formal, legal mechanism such as filing for a protective order, divorce or custody) as the demarcation for this concept analysis rather than emotional separation described by Dekerseredy et al. (2017). It is the physical or legal separation that explicitly leads to post-separation abuse behaviours.

*Legal abuse* includes ‘custody stalking’ (Elizabeth, 2017), the attempt and threats to ‘take children away’ via custody proceedings, instigating frivolous lawsuits or other system-related manipulations (Bancroft et al., 2002; Galántai et al., 2019; Gutowski & Goodman, 2020; Hines et al., 2015; Miller & Smolter, 2011; Silverman et al., 2004). Legal abuse may include litigation tactics that shift blame to victims and reduce their credibility (Harsey & Freyd, 2020).

*Economic abuse* includes withholding access to resources (child support), medical expenses for children or interfering with the survivor's ability to work (Bell et al., 2007; Brownridge, 2006; Cleak et al., 2018). Interferences with employment can include creating chaos with access schedules to produce childcare hardships, causing conflict at the survivor's place of employment, or involving the employer in litigation.

*Threats and endangerment to children* includes threats to harm or kidnap children, refusal to return children, physical or sexual abuse of children, medical/psychological neglect or putting children in age inappropriate settings such as leaving unattended with firearms, exposing to hostile gun displays (Azrael & Hemengway, 2000), pornography or illicit drugs (Hayes, 2017).

*Isolating and discrediting* includes portraying the survivor as an unfit parent, accusing them of parental alienation (Meier, 2020), spreading rumours about their mental health (Gutowski & Goodman, 2020) or extending stalking, harassment and legal abuse to the survivor's support system. The impact of parental alienation allegations in family courts is gendered: mothers accused of parental alienation were more likely to lose custody than fathers (Meier, 2020) and judges implicitly assume mothers are the ‘gatekeepers’ of fathers' relationships with their children (Austin et al., 2013).

*Harassment and stalking* are forms of abuse designed to intimidate, create fear and exert power and control over a former partner. Behaviours include violations of protective orders or custody orders, frequent unwanted contact (Logan et al., 2008; Logan & Walker, 2004; Lynch et al., 2021; Walker et al., 2004) or using third parties to harass (Messing et al., 2020). Custody arrangements often legitimize the abusive partners' contact, providing opportunities for harassment (Wuest et al., 2006). Nearly half (42%–50%) of abusive men violate protective orders (Logan et al., 2008). A history of multiple breaches of court orders, stalking and a highly controlling ex-partner are indications of high risk of lethality for women and children (Sachmann & Johnson, 2014).

## 5 ∣ ESSENTIAL ATTRIBUTES

Walker and Avant (2019) describe essential attributes as key characteristics of the concept. Because these occur in a sociolegal context, the historical and cultural environment of gender and patriarchal norms influences the current legal context, which in turn establishes the over-arching context in which post-separation abuse occurs. Post-separation abuse is best viewed as a cumulative pattern of behaviour, rather than incident specific (Katz et al., 2020; Stark & Hester, 2019). The following essential attributes of post-separation abuse were identified: fear and intimidation; domination, power and control; intrusion and entrapment; and omnipresence (Figure 1).

### 5.1 ∣ Fear and intimidation

Intimidation manifests as psychological abuse and includes tactics such as damaging property, gaslighting and non-verbal threats such as hostile gun displays (Brownridge, 2006; Brownridge et al., 2008; Crossman et al., 2016; Hardesty & Ganong, 2006; Miller & Smolter, 2011; Stark & Hester, 2019). As part of creating a climate of fear, abusive ex-partners weaponize what means most to their former partners, which is often their children (Toews & Bermea, 2017). Threatening behaviour––and an individual's perceived sense of threat based on the pattern of past violence they have experienced––may be largely invisible and not understood by professionals involved in family court litigation (Haselschwerdt et al., 2011; Katz et al., 2020; Rivera et al., 2012; Saunders et al., 2013). This lack of understanding, and climate of fear, hampers the ability of women experiencing IPV to negotiate and obtain safe co-parenting arrangements (Cleak et al., 2018; Toews & Bermea, 2017), entrapping them to further post-separation abuse.

### 5.2 ∣ Domination, power and control

Post-separation abuse is designed to make the former partner feel powerless, and power and control is central to understanding violence towards an intimate partner (Godfrey & Robinson, 2014; Katz et al., 2020; Miller & Smolter, 2011; Stark & Hester, 2019). Domination includes coercive tactics such as technological harassment, stalking and threats, and can be underwritten by a legal system that does not take action to stop these tactics. Abusive former partners are more likely to seek sole physical and legal custody than non-abusive former partners, and are often awarded custody even with documented, substantiated and criminal convictions of IPV against the mother (Bancroft et al., 2002; Meier, 2020; Miller & Smolter, 2011; Silberg & Dallam, 2019). When abusers fight for and obtain custody, what they are often looking for is not more meaningful involvement with their children, but rather acknowledgement of their status and importance (Bancroft et al., 2002; Brown et al., 2019; Silverman et al., 2004; Slote et al., 2005). The cumulative impact of domination, power and control tactics is that mothers experiencing post-separation abuse are rendered powerless to protect their children and powerless to escape ongoing abuse (Gutowski & Goodman, 2020).

### 5.3 ∣ Intrusion and entrapment

Post-separation abuse can be thought of as relentless attacks on a former partner's autonomy that continues throughout post-separation parenting, and results in a state of ‘continuous entrapment’ (Hardesty, 2002; Katz et al., 2020; Stark & Hester, 2019). Wuest et al. (2006) identified intrusion as the primary barrier to health promotion for women following separation from an abusive partner, which was characterized by continued abuse, harassment, the costs of negotiating support and the cumulative effects of stress and abuse on women and children's health and well-being. Frequent manipulation of access schedules is an additional way perpetrators use children to create intrusion (Toews & Bermea, 2017; Zeoli et al., 2013). Intrusion diverts resources away from children and other priorities (Francia et al., 2019), and limits the ability to negotiate safety, healing and achieve long-term autonomy.

### 5.4 ∣ Omnipresence

Past experiences of violence cast a long shadow, producing a mental state where fear of the perpetrator is always present, leading to the inability to escape in time, place and space (Henze-Pedersen, 2021; Humphreys et al., 2019; Katz et al., 2020). Although a survivor may be separated in physical space, technology allows perpetrators to overcome geographical boundaries (Markwick et al., 2019; Messing et al., 2020). As a result, physical separation from an abusive partner may create neither safety, nor freedom (Katz et al., 2020). Stalking and harassing tactics, even those not reaching criminal levels, communicate that abusers can access and affect them at any time (Zeoli et al., 2013). Government sanctioned parenting-time arrangements create opportunities to force contact, and may prevent the ability to set healthy boundaries (Bendlin & Sheridan, 2019; Toews & Bermea, 2017). Abusers may use subtle behaviours that come across to others as being an ‘involved’ parent, such as creating additional excuses for contact (Nikupeteri & Laitinen, 2015), but survivors recognize these tactics as intrusion or harassment.

### 5.5 ∣ Manipulations of systems

Abusers manipulate systems to prevent formal help-seeking behaviours, exert power, force contact and financially burden survivors (Miller & Smolter, 2011). This can include litigation strategies used in response to help-seeking behaviours, such as filing for custody in response to a survivor seeking a protection order or reporting violence to police (Miller & Smolter, 2011). ‘Parental alienation’ is used as a tactic to undermine allegations of domestic violence and child maltreatment (Haselschwerdt et al., 2011; Laing, 2017; Lapierre & Côté, 2016; Meier, 2010; Meier, 2017). When there is a custody dispute, judges are less likely to grant protective orders (Rosen & O'Sullivan, 2005), and child protective services (CPS) are less likely to investigate reports of abuse (Black et al., 2021; Saini et al., 2013). Abusers can use aspects of the court process to humiliate and terrorize their former partners, often weaponizing their personal history (Miller & Smolter, 2011), including their mental and physical health. For instance, mothers who seek mental health treatment for depression or anxiety that directly stems from the abuse they experienced risk being perceived as an unfit parent, cast as psychologically unstable (Gutowski & Goodman, 2020; Watson & Ancis, 2013) and having this used against them in court proceedings (Wuest et al., 2006).

## 6 ∣ MODEL CASE AND CONTRARY CASE

In Walker and Avant's (2019) method of concept analysis, the model case (Table 1) is presented as a ‘real life’ example that demonstrates the defining attributes of post-separation abuse. In contrast, a contrary case is a clear example of what the concept certainly is not. We have illustrated both a model case and contrary case, which are amalgamations from qualitative examples in the literature. These illustrations may be helpful to better understand experiences of post-separation abuse.

## 7 ∣ ANTECEDENTS

Walker and Avant (2019) describe antecedents as the events that must occur prior to the occurrence of the concept. Antecedents to post-separation abuse include patriarchal norms, pre-separation IPV or coercive control, perpetrator characteristics, physical separation and spatiality or availability (Figure 1).

### 7.1 ∣ Patriarchal norms

Patriarchal norms create the context for post-separation abuse by men towards women through gendered notions of caregiving of children, male entitlement and gender bias in courts (Davies et al., 2009; Meier, 2020). IPV perpetration is strongly associated with men's adherence to familial patriarchal ideology (e.g. men's sense of ownership over wives and children), men's use of pornography, substance use and male peer support that endorsed violence as a means to control (DeKeseredy & Joseph, 2006).

### 7.2 ∣ Pre-separation family context of IPV and coercive control

IPV during the relationship is the strongest predictor of post-separation abuse (Ellis et al., 2021; Galántai et al., 2019). Other family factors that can be considered antecedents for post-separation abuse include marriage or cohabitation, sharing children and separation. Violence that occurred during a relationship continues to influence the perception of the power of the abuser because the survivor knows what the abuser is capable of (Toews & Bermea, 2017).

### 7.3 ∣ Spatiality and availability

Because physical proximity may be limited in the post-separation context, batterers devise tactics that take advantage of their former partner's availability. For example, court mandated periods such as court appearances and custody or visitation exchanges of children offer opportunities where the survivor is mandated to be available in the presence of the abuser. Batterers may also deploy other tactics that circumvent physical barriers such as electronic harassment (Markwick et al., 2019; Messing et al., 2020).

### 7.4 ∣ Perpetrator characteristics

Characteristics of individuals who perpetrate post-separation abuse include narcissism, lack of empathy, jealousy, vulnerability, high dependence (Ellis, 2017) and blame-shifting behaviours (Brownridge, 2006; Hardesty, 2002; Harsey & Freyd, 2020; Sachmann & Johnson, 2014). Perpetrators often have a charming public image, making it difficult for survivors to seek help and be believed and contributes to manipulation of systems (Katz et al., 2020). Additional characteristics of abusive partners include high levels of denigration and disparagement, lack of insight or attention into how their own parenting impacts children and a tendency to place sole blame for problems in the family on the survivor (Bancroft et al., 2002; Katz et al., 2020; Thompson-Walsh et al., 2018; Turhan, 2021).

## 8 ∣ CONSEQUENCES

According to Walker and Avant (2019), consequences are the events and outcomes that occur as a result of the concept. Consequences of post-separation abuse include lethality, health consequences, economic deprivation and institutional violence and betrayal (Figure 1).

### 8.1 ∣ Lethality

The most severe consequence of post-separation abuse is intimate partner homicide. Maternal and child deaths are associated with custody disputes and contact arrangements (Holt, 2015; Kernic et al., 2005). The combination of physical and legal separation created the greatest risk of murder by an intimate partner (Campbell et al., 2007; Ellis, 2017; Wilson & Daly, 1993). In addition, Campbell et al. (2009) found that a partner who was highly controlling increased significantly the risk of homicide for female partners who had left their abusers. The first 3 months and the first year following separation are the most lethal, with the risk declining over time (Campbell et al., 2003; Campbell et al., 2007).

### 8.2 ∣ Health consequences

Ongoing post-separation abuse has devastating health consequences for children and adults who experience violence. Longlasting negative emotional and mental health sequelae for women from post-separation abuse includes PTSD, depression and anxiety (Crosse & Millar, 2017; Ellis et al., 2021; Estefan et al., 2016). Survivors experience adverse physical health consequences relating to both physical injury and somatization of stress, including traumatic brain injury (Valera et al., 2021), chronic pain, gastrointestinal symptoms, reproductive health, neuroendocrine alterations and epigenetic changes (Ford-Gilboe et al., 2011; Ford-Gilboe et al., 2015). A history of stalking is associated with increased severity of post-traumatic stress symptoms, even after controlling for partner abuse (Fleming et al., 2012). Moreover, the sense of powerlessness that is reinforced for IPV survivors who encounter indifference or hostility to their help-seeking behaviours reinforces emotional trauma (Buckley et al., 2011). Denying children access to medications or needed healthcare, especially mental health, is another consequence of post-separation abuse (Silberg & Dallam, 2019; Toews & Bermea, 2017). In addition to IPV exposure, children may experience neglect or physical or sexual abuse (Holt, 2020), with 30%–77% of families experiencing IPV also experiencing child maltreatment (Edleson, 1999; Silberg & Dallam, 2019).

### 8.3 ∣ Economic deprivation

IPV is associated with employment instability, childcare and housing stressors causing material hardship (Bell et al., 2007; Estefan et al., 2016). Economic deprivation can be caused by a batterer's use of court action to exhaust financial resources of their former partner, rendering them bankrupt and financially destitute (Crosse & Millar, 2017; Miller & Smolter, 2011; Toews & Bermea, 2017). In addition to the cost of legal representation, legal abuse can impact economic well-being including increased childcare burdens, lost productivity and transportation difficulties (Gutowski & Goodman, 2020; Miller & Smolter, 2011). IPV survivors who are fearful of abuse often lower demands for child support, which results in trading safety for long-term financial well-being of their children (Hardesty, 2002). Qualitative research has highlighted that many survivors feel that they ‘gave up everything’ to get out of abusive marriages (Toews & Bermea, 2017).

### 8.4 ∣ Institutional violence and betrayal

Institutional violence may take the form of loss of custody of one's children, lack of investigation and lack of justice (Gutowski & Goodman, 2020). It is precisely when survivors seek out formal sources of help that they come into contact with institutions like family court. However, mothers experiencing IPV often face a catch 22: they risk losing custody to child protective services or being criminalized for failure-to-protect their children, or they risk losing custody to their abuser for being seen as alienating or unwilling to co-parent (Meier, 2020; Saunders & Oglesby, 2016).

## 9 ∣ DISCUSSION

### 9.1 ∣ Empirical referents

Empirical referents are the measurement tools that demonstrate the occurrence of concept (Walker & Avant, 2019). The study of IPV has faced persistent definitional and measurement dilemmas (Crossman et al., 2016), and no measurement tool exists that measures the complexity of long-term, ongoing abuse experiences following separation from an abusive partner and co-parent (Cleak et al., 2018). None of the existing measures include aspects of legal abuse, using children or threats to take custody and only the Danger Assessment (Campbell et al., 2009) includes threats of harm to children (Jaffe et al., 2012). Studies reviewed for this concept analysis used instruments including Partner Abuse Scale (Attala et al., 1994), Revised Conflict Tactics Scale (Straus et al., 1996), Abuse Assessment Screen (Parker & McFarlane, 1991) and stalking screening tools such as the NVAWS (Tjaden & Thoennes, 2000). Other empirical referents used in the post-separation context that most closely capture experiences of post-separation abuse include Women's Experience of Battering (WEB) (Smith et al., 1995), HARASS (Sheridan, 2001) and the Danger Assessment (Campbell et al., 2009).

### 9.2 ∣ Implications for nursing

Nurses play an important role in supporting individuals experiencing IPV and their children from ongoing intrusive consequences (Broughton & Ford-Gilboe, 2017; Wuest et al., 2006) and can take action to address post-separation abuse (Table 2). Survivors of IPV are high users of health services and their children have high health and developmental needs (Abdulmohsen Alhalal et al., 2012; Campbell et al., 2018). Adult survivors and their children may be trapped in a web of fear and violence, and the protective parent's opportunities to safeguard children may be limited or nonexistent because of structural barriers such as court orders regulating shared parenting.

Women with children who leave abusive relationships face numerous inhibitors to safety and health, including continued abuse, heightened risk for lethality, desperate need for financial resources and the risk of being separated from their children through the family court system (Abdulmohsen Alhalal et al., 2012). Controlling and threatening, but non-physically violent, behaviours have rarely been viewed as violence by policymakers, law enforcement and the legal system (Crossman et al., 2016; Stark & Hester, 2019). Yet our biology adapts to living in threatening environments, and children are especially sensitive to threats in their environment. Both exposure to IPV and parental separation or divorce are two adverse childhood experiences (ACEs) that are linked in a dose–response relationship to adverse health consequences through the lifespan (Felitti et al., 1998). Mitigation of these harms is needed through ongoing nursing interventions.

Nurses can provide anticipatory guidance for women and children experiencing post-separation abuse, help them with assessing their risk of lethal or near lethal IPV such as with the Danger Assessment (Campbell et al., 2009), safety planning such as myPlan Safety App (Glass et al., 2010; Glass et al., 2017) and identifying resources to help cope. Nurses and other healthcare professionals play an important role in advocating for children to receive needed health and developmental services, including counselling. Nurses and other healthcare providers should document information in the child's medical record; nurses may need to report to Child Protective Services (CPS) on abuse and neglect, including medical neglect.

Little is known about how firearms are used for intimidation in the context of post-separation abuse (Azrael & Hemengway, 2000). Given the increased risk of lethality in the post-separation context, firearm safety is an important consideration. Nurses should ask about and document access to firearms in each parent's home and provide instruction and guidance on safe storage behaviours. Safe storage of firearms has been shown to reduce injuries and fatalities to children, including homicides and suicides (Azad et al., 2020).

Nurses can use a strength-based approach to educate and reassure mothers who are experiencing post-separation abuse about the healing power of safe, supportive and nurturing relationships for children (CDC, 2019). Cultivating positive childhood experiences and parent–child connection can be a powerful source of healing for children and mitigate the harms they are experiencing from ongoing post-separation abuse (Bethell, Gombojav, & Whitaker, 2019; Bethell, Jones, et al., 2019).

Healthcare professionals need to be aware of the ways in which family court judicial decisions can act as a barrier to the health and safety for women and children exposed to IPV in the post-separation context. The criminal legal system has been examined for exacerbating health disparities, but the same attention has not been placed on the civil legal system, despite the family court's role as a determinant of children's health outcomes by regulating the child's environment. Judges have wide discretion in crafting orders and can implement significant guardrails to protect individuals exposed to IPV from further violence and harassment. Supervised visitation and/or exchanges, and other provisions to reduce risk such as refraining from alcohol and other substances during visitation may help keep mothers and children safe from abuse, but given the costs associated may not be available in all jurisdictions and are no panacea (Pond & Morgan, 2008; Spearman et al., 2022). Nurses can advocate for policies and judicial training that is trauma-informed and promotes understanding the complexities and nuances of the ways in which abusers continue to harass their former partners when they share children (Eilers, 2019).

### 9.3 ∣ Limitations

A limitation to this concept analysis is a lack of quantitative data on the incidence, prevalence, severity and health consequences of post-separation abuse. Co-parenting conflict has been studied separately from co-parenting in the context of IPV (Hardesty et al., 2019), or they have been lumped together making it difficult to differentiate post-separation abuse from non-abusive conflict. Given the implications of fear and threat on children's neurodevelopment (McLaughlin et al., 2014), understanding the implications of post-separation abuse on children's health and well-being is an important area for future study. Little empirical data exists on how post-separation abuse may change over time (Hardesty et al., 2017), and chronicity and frequency of exposure to post-separation abuse are factors that need to be explored. Another significant limitation is the lack of attention to diverse populations in the studies reviewed for this concept analysis. This is a significant gap that needs to be addressed to understand the intersectional vulnerabilities in the post-separation context for those with historically marginalized and minoritized identities. Most studies reviewed were from high-income countries, and reflected heterosexual partnerships. Future work should investigate how post-separation abuse varies across legal jurisdictions, across gender and across same sex partnerships. Because of the financial resources required to access the civil legal system in the United States and elsewhere, future work should also address how post-separation abuse varies across socio-economic circumstances.

## 10 ∣ CONCLUSION

There is a need to measure post-separation abuse to understand its incidence and prevalence and to develop interventions to promote healing, safety and well-being. There is a need for more widespread knowledge about intersections of health, law and domestic violence so nurses are better positioned to advocate for children and IPV survivors (Anselmi, 2011). Separation from an abusive partner has been identified as an ongoing process or transition (Dekerseredy et al., 2017), and the middle range theory of Experiencing Transitions (Meleis et al., 2000) could be useful to guide future nursing research in this field.

## Figures and Tables

**TABLE 1:** Model and contrary cases of post-separation abuse

Model case
A is a stay-at-home mother of two children. Over the years, she experienced a pattern of emotional abuse and threats from her spouse. When her 6-year-old son told her that his dad had grabbed him by his neck, shoved his head into the wall, and he had an accident because he was scared, A fled their home. She reported the abuse to child protective services as required by law; however, her husband immediately filed for sole custody alleging that A had kidnapped the children when she fled with them, was psychologically abusive to the children, and was mentally unfit. A's estranged partner continued to show up unannounced, sent dozens of emails and texts to her each day, and hired private investigators to follow her. During transitions of the children between households, A's ex-partner would point to his car where she knew he stored his gun, and remind her that she better stay in line.
During his parenting-time, he often refused to let the children communicate with A. He constantly told the children lies about her and got them to promise not to tell her. As A tried to obtain employment, her ex-partner called and harassed her at work, subpoenaed her employer for court proceedings, and frequently dropped the children off early from his visitation periods resulting in an inability for A to obtain last minute childcare. She experienced multiple flat tires, causing her to miss work and the frequent repairs caused a financial hardship. Despite a court order to pay child support, A's ex-partner frequently withheld child support despite an ability to pay, causing A to struggle with housing insecurity. Because of mounting legal fees to maintain custody of her children, A has filed bankruptcy. A tried to minimize conflict by setting boundaries for communication and interaction, but A's estranged partner construed these efforts as hostile with the intent of alienating him from their children and took her back to court. To avoid further litigation she could not afford, and because she feared losing additional access to her children, A increasingly agreed to her ex-partners demands even though she feared for the safety and well-being of their children. Although her children were distressed and had special health and developmental needs, A is unable to obtain healthcare for her children because her ex-partner refuses to consent and withholds their health insurance.
Contrary case
The following example is provided to illustrate what post-separation abuse is not. B has two children and was married for 10 years, working part-time since she had children. Although the separation was at times stressful with charged emotions and heated arguments, there was no history of IPV or coercive control. Both parents value the others' contributions to parenting. B's ex-partner supported her efforts in obtaining full time employment post-separation, and was flexible in designing a co-parenting schedule that worked for both of them. B's ex-partner acknowledged they were no longer good for each other, but he spoke of valuing her contributions as a mother, and that they were in this together. B and her ex-partner were working with a parent educator to learn how to set healthy boundaries with each other following divorce, and work together on shared values for how to raise their children. While they continue to have disagreements as they work through their anger and sadness, neither parent is fearful of the other, there are no safety concerns for the children, and they both made a commitment to keep all communication respectful and uphold agreements they made.

**TABLE 2:** Implications for nursing

Ten ways nurses can address post-separation abuse
Nurses should elicit more information from parents around the nature of high conflict custody cases, so that conflict is not conflated with IPV/child maltreatment and to ensure that appropriate interventions are applied. Nurses can ask questions such as *‘How does the relationship with your coparent make you feel?’* which may elicit more disclosure about the patterns of abuse experienced than questions such as ‘Do you feel safe at home?’. Nurses can administer risk assessment tools, such as the Danger Assessment, or help women use tools such as the MyPlan Safety App, which provides feedback to the user about her risk for lethal violence, provides assistance with setting priorities for safety, and creates a personalized safety plan. Nurses can connect survivors and their children with needed resources, including domestic violence advocacy organizations, including those that offer legal aid, and organizations such as Family Justice Centres that provide wrap around services. Some states and jurisdictions may have address confidentiality programmes and other services that can increase safety for survivors. Nurses play an important role in advocating for needed health and developmental services for children, which may be especially important in cases when a non-offending parent has lost legal custody. Documenting medical neglect (a child needing services that are not obtained) by a parent who is perpetrating abuse against the survivor and/or child may be helpful for appeals and or future attempts of the non-offending parent to regain custody. Nurses should document information in the child's medical record of the types of abuse, harassment, stalking and legal abuse that women and their children are experiencing and may need to report to Child Protective Services. Nurses should assess for the presence of firearms in either household and educate parents and children about safe gun storage. If guns are present, nurses should assess for whether mothers or children have experienced hostile gun displays. Nurses encourage mothers to ask for safe storage provisions or the removal of firearms in custody orders. Obtaining these provisions are important, as they may also provide an additional avenue to seek legal protection for violations of safe storage provisions without prosecution. Nurses can employ a strength-based approach and educate parents on the importance of safe, stable and nurturing relationships, and how focusing on positive childhood experiences and parent–child connection can mitigate some harms children experience. Nurses can play an important role in advocacy work, advocating for trauma-informed training for judges, custody evaluators and other professionals in the family court system on the nuances of domestic violence, adverse childhood experiences and the health implications for mothers and children.

## Data Availability

The data that supports the findings of this study are available in the supplementary material of this article

## APPENDIX 1

### Articles Identified for Post-Separation Abuse Concept Analysis

**Table:**

Author	Title	Country	Discipline	Article type/ methodology
Abdulmohsen Alhalal et al. (2012)	Identifying factors that predict women's inability to maintain separation from an abusive partner.	Canada	Nursing	Quantitative
Anderson and Saunders (2003)	Leaving an abusive partner: an empirical review of predictors, the process of leaving, and psychological well-being.	Multiple	Social work/Sociology	Literature review
Anselmi (2011)	Legal File. Domestic violence and its implications on child abuse.	US	Nursing/Law	Case study Commentary
Austin et al. (2013)	Bench Book for Assessing Parental Gatekeeping in Parenting Disputes: Understanding the Dynamics of Gate Closing and Opening for the Best Interests of Children.	US	Law	Bench book
Beck et al. (2013)	Patterns of intimate partner violence in a large, epidemiological sample of divorcing couples.	US - Arizona	Psychology	Quantitative
Bemiller (2008)	When battered mothers lose custody: a qualitative study of abuse at home and in the courts.	US – Ohio	Social work/Sociology	Qualitative
Bendlin and Sheridan (2019)	Risk Factors for Severe Violence in Intimate Partner Stalking Situations: An Analysis of Police Records	Australia	Psychology	Quantitative
Black et al. (2021)	The intersection of child welfare, intimate partner violence and child custody disputes: secondary data analysis of the Ontario incidence study of reported child abuse and neglect.	Canada	Social work/Sociology	Quantitative
Broughton and Ford-Gilboe (2016)	Predicting family health and well-being after separation from an abusive partner: role of coercive control, mother's depression and social support.	Canada	Nursing	Quantitative
Brownridge (2006)	Violence against women post-separation	Multiple	Social work/Sociology	Literature Review
Brownridge et al. (2008)	The elevated risk for non-lethal post-separation violence in Canada: a comparison of separated, divorced, and married women.	Canada	Social work/Sociology	Quantitative
Bruno (2018)	Financial oppression and post-separation child positions in Sweden.	Sweden	Social work/Sociology	Qualitative
Buckley et al. (2011)	‘Like waking up in a Franz Kafka novel’: service users' experiences of the child protection system when domestic violence and acrimonious separations are involved.	Ireland	Social work/Sociology	Qualitative
Campbell et al. (2007)	Intimate partner homicide: review and implications of research and policy.	US	Nursing	Literature review
Campbell et al. (2003)	Risk factors for femicide in abusive relationships: results from a multisite case control study.	US	Nursing/Public Health/Medicine	Mixed methods
Carroll (2000)	When domestic violence leaves home. It can and does invade the workplace.	US	Nursing	Commentary
Cleak et al. (2018)	Screening for Partner Violence Among Family Mediation Clients: Differentiating Types of Abuse.	Australia	Social Work/Sociology	Mixed methods
Cohen et al. (2002)	Interactional effects of marital status and physical abuse on adolescent psychopathology.	US – New York	Medicine (Psychiatry)	Quantitative
Cramp and Zufferey (2021)	The Removal of Children in Domestic Violence: Widening Service Provider Perspectives.	Australia	Social Work/Sociology	Qualitative
Crosse and Millar (2017)	Irish Women's Ongoing Experiences of Domestic Abuse in Cases of Separation and Divorce.	Ireland	Social Work/Sociology	Qualitative
Crossman et al. (2016)	“He Could Scare Me Without Laying a Hand on Me”: Mothers' Experiences of Nonviolent Coercive Control During Marriage and After Separation.	US	Family Science	Qualitative
Davies et al. (2008)	Gender inequality and patterns of abuse post leaving.	Canada	Social work/Nursing	Mixed methods
Davis (2002)	Leave-taking experiences in the lives of abused women.	US – Pennsylvania	Nursing	Qualitative
DeKeseredy and Joseph (2006)	Separation and/or divorce sexual assault in rural Ohio: preliminary results of an exploratory study.	US-Ohio	Criminology/Sociology	Mixed methods
DeKeseredy and Schwartz (2008)	Separation/divorce sexual assault in rural Ohio: survivors' perceptions.	US-Ohio	Criminology/Sociology	Qualitative
Drozd and Olesen (2010)	Abuse and alienation are each real: a response to a critique by Joan Meier.	US	Law	Commentary
Elizabeth et al. (2012)	The gendered dynamics of power in disputes over the postseparation care of children.	Australia	Social Work/Sociology	Qualitative
Elizabeth (2017)	Custody Stalking: A Mechanism of Coercively Controlling Mothers Following Separation	Australia	Social Work/Sociology	Qualitative
Ellis (1987)	Post-separation woman abuse: the contribution of lawyers as “barracudas,” “advocates,” and “counsellors.”	Canada	Social Work/Sociology	Typology
Ellis and Dekeseredy (1997)	Rethinking estrangement, interventions, and intimate femicide.	Canada	Social Work/Sociology	Theory
Ellis et al. (2021)	Effects of Historical Coercive Control, Historical Violence, and Lawyer Representation on Post-Separation Male Partner Violence Against Mother Litigants Who Participated in Adversarial Family Court Proceedings.	Canada	Social Work/Sociology	Quantitative
Ellis and Wight (1997)	Estrangement, interventions, and male violence toward female partners.	Canada	Social Work/Sociology	Literature review
Ellis (2017)	Marital Separation and Lethal Male Partner Violence.	Canada	Social Work/Sociology	Literature review and theory
Eriksson and Hester (2001)	Violent men as good-enough fathers? A look at England and Sweden.	England; Sweden	Social Work/Sociology	Commentary
Estefan et al. (2016)	Depression in Women Who Have Left Violent Relationships.	US – Florida	Public health	Quantitative
Faller (2016)	Commentary on the American Professional Society on the Abuse of Children's position paper on allegations of child maltreatment and intimate partner violence in divorce/parental relationship dissolution.	US	Social Work/Sociology	Commentary
Feresin et al. (2018)	Family Mediation in Child Custody Cases and the Concealment of Domestic Violence.	Italy	Social Work/Sociology/Psychology/Law	Qualitative
Fields (2008)	Getting beyond “what did she do to provoke him?”: comments by a retired judge on the special issue on child custody and domestic violence.	US	Law	Commentary
Fleming et al. (2012)	Intimate partner stalking victimization and posttraumatic stress symptoms in post-abuse women.	US	Psychology	Quantitative
Forssell and Cater (2015)	Patterns in Child-Father Contact after Parental Separation in a Sample of Child Witnesses to Intimate Partner Violence.	Sweden	Law/Social Work/Sociology	Quantitative
Francia et al. (2019)	Addressing family violence post separation: mothers and fathers' experiences from Australia.	Australia	Social Work/Sociology /	Qualitative
Galántai et al. (2019)	Children Exposed to Violence: Child Custody and its Effects on Children in Intimate Partner Violence Related Cases in Hungary.	Hungary	Social Work/Sociology /	Mixed methods
Geffner and Mueller (2015)	Introduction to the Special Issue on Intimate Partner Violence and Abuse: Issues to Consider in Child Custody Evaluations.	US	Psychology	Commentary
Gray et al. (2016)	‘I'm Working Towards Getting Back Together’: Client Accounts of Motivation Related to Relationship Status in Men's Behaviour Change Programmes in New South Wales, Australia.	Australia	Social Work/Sociology /	Qualitative
Gutowski and Goodman (2020)	“Like I'm Invisible”: IPV Survivor-Mothers' Perceptions of Seeking Child Custody through the Family Court System.	US – Massachusetts	Psychology	Qualitative
Hamby et al. (2010)	The overlap of witnessing partner violence with child maltreatment and other victimizations in a nationally representative survey of youth.	US	Psychology	Quantitative
Hans et al. (2014)	The effects of domestic violence allegations on custody evaluators' recommendations.	US	Family Science	Qualitative
Hardesty (2002)	Separation assault in the context of postdivorce parenting: an integrative review of the literature.	Multiple	Family Science	Literature Review
Hardesty et al. (2016)	Marital violence and coparenting quality after separation.	US – Midwest	Family Science	Quantitative
Hardesty and Ganong (2006)	How women make custody decisions and manage co-parenting with abusive former husbands.	US	Family Science	Qualitative
Hardesty et al. (2017)	Coparenting relationship trajectories: Marital violence linked to change and variability after separation.	US – Midwest	Family Science	Quantitative
Hardesty et al. (2019)	Relationship dynamics and divorcing mothers' adjustment: Moderating role of marital violence, negative life events, and social support.	US – Midwest	Family Science	Quantitative
Hardesty et al. (2012)	An Integrative Theoretical Model of Intimate Partner Violence, Coparenting After Separation, and Maternal and Child Well-Being.	US	Family Science	Theory development
Harrison (2008)	Implacably hostile or appropriately protective? Women managing child contact in the context of domestic violence.	United Kingdom	Social Work/Sociology	Qualitative
Haselschwerdt et al. (2011)	Custody Evaluators' Beliefs About Domestic Violence Allegations During Divorce: Feminist and Family Violence Perspectives.	US	Family Science	Qualitative
Hassouneh-Phillips (2001)	American Muslim women's experiences of leaving abusive relationships.	US	Nursing	Qualitative
Hayes (2017)	Indirect Abuse Involving Children During the Separation Process.	US	Criminology	Quantitative
Hayes (2012)	Abusive Men's Indirect Control of Their Partner During the Process of Separation.	US	Criminology	Quantitative
Henze-Pedersen (2021)	The Ghost of Violence: The Lived Experience of Violence After the Act	Denmark	Social Work/Sociology	Qualitative
Hines et al. (2015)	A self-report measure of legal and administrative aggression within intimate relationships.	US	Social Work/Sociology	Qualitative synthesis/literature review
Hing et al. (2021)	Impacts of Male Intimate Partner Violence on Women: A Life Course Perspective.	Australia	Nursing/Public Health	Qualitative
Holt (2017)	Domestic Violence and the Paradox of Post-Separation Mothering.	United Kingdom	Social Work/Sociology	Qualitative
Holt (2015)	Post-separation Fathering and Domestic Abuse: Challenges and Contradictions.	United Kingdom	Social Work/Sociology	Mixed methods
Holt (2020)	Domestic Abuse and Post-Separation Contact: Promoting Evidence and Informed Practice.	United Kingdom	Social Work/Sociology	Commentary
Humphreys et al. (2019)	More present than absent: Men who use domestic violence and their fathering.	Australia	Social Work/Sociology	Mixed methods
Ingrids (2014)	Category work in courtroom talk about domestic violence: Gender as an interactional accomplishment in child custody disputes.	Sweden	Psychology	Qualitative
Jaffe and Crooks (2004)	Partner violence and child custody cases: a cross national comparison of legal reforms and issues.	US, Canada, Australia, New Zealand	Psychology	Literature review
Jaffe et al. (2009)	A framework for addressing allegations of domestic violence in child custody disputes.	Canada	Psychology	Theoretical framework
Jaffe et al. (2008)	Custody disputes involving allegations of domestic violence: Toward a differentiated approach to parenting plans	Canada	Psychology	Theoretical framework
Johnson (2005)	Apples and oranges in child custody disputes: intimate terrorism vs. situational couple violence.	US	Social Work/Sociology	Commentary
Jones and Vetere (2017)	‘You just deal with it. You have to when you've got a child’: A narrative analysis of mothers' accounts of how they coped, both during an abusive relationship and after leaving	United Kingdom, Norway	Psychology	Qualitative
Kan et al. (2012)	Intimate Partner Violence and Coparenting Across the Transition to Parenthood.	US	Psychology	Quantitative
Katz et al. (2020)	When Coercive Control Continues to Harm Children: Post-Separation Fathering, Stalking and Domestic Violence	United Kingdom/Finland	Social Work/Sociology	Qualitative
Kernic et al. (2005)	Children in the crossfire: child custody determinations among couples with a history of intimate partner violence.	US – Washington State	Public health	Quantitative
Khaw et al. (2021)	“The System Had Choked Me Too”: Abused Mothers' Perceptions of the Custody Determination Process That Resulted in Negative Custody Outcomes.	US – Midwest and West Coast	Family Science	Qualitative
Khaw and Hardesty (2015)	Perceptions of boundary ambiguity in the process of leaving an abusive partner.	US – Midwest	Family Science	Qualitative
Kieffer and Turell (2011)	Child Custody and Safe Exchange/Visitation: An Assessment of Marginalized Battered Parents' Needs.	US	Social work/Sociology	Mixed methods
Kolsky and Gee (2021)	Coparenting Quality Mediates the Association Between Intimate Partner Violence and Child Behavior Problems in Low-income, Racial and Ethnic Minority Families.	US – Midatlantic	Psychology	Quantitative
Kong (2021)	Beyond ‘Safeguarding’ and ‘Empowerment’ in Hong Kong: Towards a Relational Model for Supporting Women Who Have Left their Abusive Partners.	Hong Kong	Social work/Sociology	Qualitative
Laing (2017)	Secondary Victimization: Domestic Violence Survivors Navigating the Family Law System.	Australia	Social work/Sociology	Qualitative
Lambert (2015)	Introduction to the Special Issue on Attitudes and Current Research Concerning Intimate Partner Violence: Issues for Child Custody.	US	Psychology	Commentary
Lapierre and Côté (2016)	Abused women and the threat of parental alienation: Shelter workers' perspectives.	Canada	Social work/Sociology	Mixed methods
Logan and Walker (2004)	Separation as a risk factor for victims of intimate partner violence: beyond lethality and injury: a response to Campbell.	US	Psychology	Commentary
Logan et al. (2003)	Divorce, custody, and spousal violence: a random sample of circuit court docket records.	US – Kentucky	Psychology/Public Health	Quantitative
Logan et al. (2008)	Factors associated with separation and ongoing violence among women with civil protective orders.	US	Psychology	Mixed methods
Lynch et al. (2021)	Coercive Control, Stalking, and Guns: Modeling Service Professionals' Perceived Risk of Potentially Fatal Intimate Partner Gun Violence.	US	Criminology/Psychology	Quantitative
Lyons et al. (2021)	Risk Factors for Child Death During an Intimate Partner Homicide: A Case–Control Study	US	Public health	Quantitative
Markwick et al. (2019)	Technology and Family Violence in the Context of Post-Separated Parenting.	Australia, multiple	Social work/Sociology	Literature review
McMurray (1997)	Violence against ex-wives: anger and advocacy.	Australia	Nursing	Qualitative
Mechanic et al. (2000)	The impact of severe stalking experienced by acutely battered women: an examination of violence, psychological symptoms and strategic responding.	US	Psychology	Quantitative
Meier (2010)	Getting real about abuse and alienation: a critique of Drozd and Olesen's decision tree.	US	Law	Commentary
Meier (2015)	Johnson's Differentiation Theory: Is It Really Empirically Supported?	US	Law	Commentary
Meier (2020)	U.S. child custody outcomes in cases involving parental alienation and abuse allegations: what do the data show?	US	Law	Quantitative
Meyer and Stambe (2020)	Mothering in the Context of Violence: Indigenous and Non-Indigenous Mothers' Experiences in Regional Settings in Australia.	Australia	Criminology/Social Work	Qualitative
Miller and Manzer (2021)	Safeguarding Children's Well-Being: Voices From Abused Mothers Navigating Their Relationships and the Civil Courts.	US	Sociology	Qualitative
Miller and Smolter (2011)	Paper Abuse: When All Else Fails, Batterers Use Procedural Stalking.	US	Sociology	Qualitative
Morrison (2015)	‘All Over Now?’ The Ongoing Relational Consequences of Domestic Abuse through Children's Contact Arrangements.	United Kingdom	Sociology	Qualitative
Nielsen et al. (2016)	Exploring Variations Within Situational Couple Violence and Comparisons With Coercive Controlling Violence and No Violence/No Control.	US – Midwest	Family Science	Quantitative
Nikupeteri and Laitinen (2015)	Children's Everyday Lives Shadowed by Stalking: Post separation Stalking Narratives of Finnish Children and Women.	Finland	Sociology	Qualitative
Nikupeteri (2017)	Professionals' critical positionings of women as help-seekers: Finnish women's narratives of help-seeking during post-separation stalking	Finland	Sociology	Qualitative
Ornstein and Rickne (2013)	When does intimate partner violence continue after separation?	Sweden	Economics	Quantitative
Pagelow (1993)	Justice for victims of spouse abuse in divorce and child custody cases.	US	Sociology	Commentary
Pedersen et al. (2013)	Explaining aboriginal/non-aboriginal inequalities in postseparation violence against Canadian women: application of a structural violence approach.	Canada	Medicine/Public Health	Quantitative
Pitman (2017)	Living with Coercive Control: Trapped within a Complex Web of Double Standards, Double Binds and Boundary Violations.	Australia	Social Work/Sociology	Qualitative
Pond and Morgan (2008)	Protection, manipulation or interference with relationships? Discourse analysis of New Zealand lawyers' talk about supervised access and partner violence.	New Zealand	Psychology	Qualitative
Rennison et al. (2013)	Intimate relationship status variations in violence against women: urban, suburban, and rural differences.	US	Political science/Criminology	Quantitative
Rezey (2020)	Separated Women's Risk for Intimate Partner Violence: A Multiyear Analysis Using the National Crime Victimization Survey.	US	Criminology	Quantitative
Rivera et al. (2018)	A Longitudinal Examination of Mothers' Depression and PTSD Symptoms as Impacted by Partner-Abusive Men's Harm to Their Children.	US	Psychology/Criminology	Quantitative
Rivera et al. (2012)	Abused Mothers' Safety Concerns and Court Mediators' Custody Recommendations.	US	Psychology/Criminology	Mixed Methods
Rosen and O'Sullivan (2005)	Outcomes of custody and visitation petitions when fathers are restrained by protection orders: the case of the New York family courts.	US	Law	Quantitative
Saini et al. (2013)	Child Custody Disputes within the Context of Child Protection Investigations: Secondary Analysis of the Canadian Incident Study of Reported Child Abuse and Neglect.	Canada	Social work/sociology	Quantitiatve
Saunders (1994)	Child custody decisions in families experiencing woman abuse.	US	Social work/sociology	Literature review
Saunders et al. (2013)	Factors associated with child custody evaluators' recommendations in cases of intimate partner violence.	US	Social work/Sociology/Psychology	Quantitative
Saunders (2015)	Research Based Recommendations for Child Custody Evaluation Practices and Policies in Cases of Intimate Partner Violence.	US	Social work/Sociology	Literature review
Saunders (2007)	Child Custody and Visitation Decisions in Domestic Violence Cases: Legal Trends, Risk Factors, and Safety Concerns	US	Social work/Sociology	Literature review
Shalansky et al. (1999)	Abused women and child custody: the ongoing exposure to abusive ex-partners.	Canada	Nursing	Qualitative
Shaw (2017)	Commentary regarding parenting coordination in cases of high conflict disputes.	US	Psychology	Commentary
Shepard and Hagemeister (2013)	Perspectives of Rural Women: Custody and Visitation With Abusive Ex-Partners.	US – Midwest	Social Work/Sociology	Qualitative
Shetty and Edleson (2005)	Adult domestic violence in cases of International Parental Child Abduction.	US/International	Social Work/Sociology/Public Policy/Law	Literature review
Silverman et al. (2004)	Public health matters. Child custody determinations in cases involving intimate partner violence: a human rights analysis	US – Massachusetts	Public health/Law	Qualitative
Slote et al. (2005)	Battered mothers speak out: participatory human rights documentation as a model for research and activism in the United States.	US – Massachusetts	Public health/Law	Qualitative
Louis et al. (2017)	How mothers perceive their own domestic violence victimization and how it impacts their children.	Trinidad & Tobago	Social Work/Sociology	Qualitative
Stark and Hester (2019)	Coercive Control: Update and Review.	US/United Kingdom	Social Work/Sociology	Literature review
Thiara and Humphreys (2017)	Absent presence: the ongoing impact of men's violence on the mother–child relationship.	Australia	Social Work/Sociology	Qualitative
Thompson-Walsh et al. (2018)	Are we in this Together? Post-Separation Co-Parenting of Fathers with and without a History of Domestic Violence.	Canada	Psychology	Qualitative
Toews and Bermea (2017)	“I Was Naive in Thinking, ‘I Divorced This Man, He Is Out of My Life’”: A Qualitative Exploration of Post-Separation Power and Control Tactics Experienced by Women.	US	Family Science	Qualitative
Toews and Bermea (2017)	Male-initiated partner abuse during marital separation prior to divorce.	US	Family Science	Quantitative
Tubbs (2010)	African American women's perspectives of shared parenting after dissolution of a violent relationship.	US	Social Work/Psychology	Qualitative
Turhan (2021)	Safe Father-Child Contact Postseparation in Situations of Intimate Partner Violence and Positive Fathering Skills: A Literature Review	Multiple settings	Social Work/Sociology	Literature review
Vatnar and Bjørkly (2012)	Does Separation or Divorce Make any Difference? An Interactional Perspective on Intimate Partner Violence with Focus on Marital Status.	Norway	Psychology	Mixed methods
Vu et al. (2014)	Divorce in the context of domestic violence against women in Vietnam.	Vietnam	Public health	Qualitative
Walker et al. (2004)	An integrative review of separation in the context of victimization: consequences and implications for women.	US/Multiple	Psychology/Nursing	Literature review
Warnecke et al. (2017)	Sheltering for Safety in Community Women With Divorce Histories.	US	Psychology	Quantitative
Watson and Ancis (2013)	Power and control in the legal system: from marriage/relationship to divorce and custody.	US	Psychology	Qualitative
Weisz and Wiersma (2011)	Does the Public Hold Abused Women Responsible for Protecting Children?	US – Michigan	Social Work/Sociology	Quantitative
Wilson and Daly (1993)	Spousal homicide risk and estrangement.	Canada, Australia, US	Psychology	Quantitative
Wooldredge and Thistlethwaite (2006)	Changing marital status and desistance from intimate assault.	US – Ohio	Criminology	Quantitative
Wuest et al. (2004)	Regenerating family: strengthening the emotional health of mothers and children in the context of intimate partner violence.	Canada	Nursing	Qualitative
Wuest et al. (2006)	Using grounded theory to generate a theoretical understanding of the effects of child custody policy on women's health promotion in the context of intimate partner violence.	Canada	Nursing	Qualitative; theory development
Zeoli et al. (2013)	Post-Separation Abuse of Women and their Children: Boundary-setting and Family Court Utilization among Victimized Mothers.	US – Midwest	Criminology/Psychology/Sociology	Qualitative
